# Child behaviour and subsequent changes in body weight, composition and shape

**DOI:** 10.1371/journal.pone.0226003

**Published:** 2019-12-19

**Authors:** Katrine G. Christensen, Sidse G. Nielsen, Nanna J. Olsen, Christine Dalgård, Berit L. Heitmann, Sofus C. Larsen

**Affiliations:** 1 Research Unit for Dietary Studies at The Parker Institute and Institute of Preventive Medicine, The Capital Region, Copenhagen, Denmark; 2 Department of Public Health, Environmental Medicine, University of Southern Denmark, Odense, Denmark; 3 The Boden Institute of Obesity, Nutrition, Exercise & Eating Disorders, The University of Sydney, Sydney, Australia; 4 Department of Public Health, University of Copenhagen, Copenhagen, Denmark; University College London, UNITED KINGDOM

## Abstract

**Objective:**

Studies have found an association between child behavioural problems and overweight, but the existing evidence for this relationship is inconsistent, and results from longitudinal studies are sparse. Thus, we examined the association between behavioural problems and subsequent changes in body mass index (BMI) and anthropometry over a follow-up period of 1.3 years among children aged 2–6 years.

**Design:**

The study was based on a total of 345 children from The Healthy Start Study; all children were healthy weight but predisposed to develop overweight. The Danish version of the Strengths and Difficulties Questionnaire (SDQ), classified as SDQ Total Difficulties (SDQ-TD) and SDQ Prosocial Behaviour (SDQ-PSB), was used to assess child behaviour. Linear regression analyses were used to examine associations between SDQ scores and subsequent change in BMI z-score, body fat percentage, waist circumference and waist-hip ratio, while taking possible confounding factors into account.

**Results:**

We found an association between SDQ-PSB and subsequent change in BMI z-score (β: 0.040 [95% CI: 0.010; 0.071, p = 0.009]). However, there was no evidence of an association between SDQ-PSB and measures of body composition or body shape.

**Conclusions:**

Among 2 to 6 years old children predisposed to overweight, the association between SDQ-scores and weight gain is either absent or marginal. The SDQ-PSB score may be associated with subsequent increases in BMI z-score, but this association does not seem driven by an increased relative fat accumulation.

## Background

Overweight in childhood is a strong independent risk factor for overweight and obesity in adulthood, which can lead to mental health problems, lifestyle diseases and premature death [1–3]. Therefore, it is essential to focus on prevention of obesity early in childhood to prevent the negative consequences [4].

To date, the vast majority of early childhood prevention strategies have focused on changing diet and physical activity habits. However, according to a 2011 Cochrane review on obesity prevention strategies, these are not effective intervention strategies themselves to prevent childhood overweight [1]. Hence, an increased focus has been directed towards the relationship between child behavioural problems and obesity [5].

Several different questionnaires that enable systematic assessment of the child's behaviour and related problems have been developed [6]. The Strengths and Difficulties Questionnaire (SDQ) is a frequently used instrument for this purpose and screens the well-being and behaviour of the child [7]. A possible association between the SDQ and body weight has been suggested in several studies. Cross-sectional studies have found a direct association between peer problems and overweight or obesity in children aged 5–6 and 12–18 years [8–10]. Moreover, among children aged 2–6 years from the Healthy Start Study, we previously found higher body mass index (BMI) z-scores among those children with behavioural difficulties above the 90^th^ percentile [11]. Similarly, Sawyer et al. found that children with overweight aged 4–5 years had slightly higher rates of mental health problems than children with normal weight [12]. Finally, Tiffin et al. found an association between obesity and externalizing and internalizing behaviour [13]. Although these studies suggest an association between SDQ and body weight, a direction of the association could not be established as all studies were cross sectional.

The association between body weight and development in SDQ has been investigated in some prospective studies, suggesting that obesity is detrimental to the child's mental development [14]. It is possible that the relationship is bidirectional, however, the association between SDQ and subsequent development in body weight and composition has generally not been studied.

Given this background, the aim of our study was to examine the associations between behaviour and subsequent changes in BMI z-score, composition and shape over a period of 1.3 years among children aged 2–6 years.

## Methods and materials

### Study population

The present study was based on data from the Healthy Start study (ClinicalTrials.gov, ID: NCT01583335). Healthy Start was designed as a randomized intervention with the aim of preventing overweight and obesity in children aged 2–6 years, who were primarily normal weight, but predisposed to overweight because of having either a high birth weight (> 4000 grams), a mother who was overweight prior to pregnancy (BMI > 28), or a mother with low SES (educational level ≤ 10 years). The intervention was family based, and strategies included optimization of diet-, physical activity- and sleep habits, and reduced stress. The average intervention time was 1.3 years. A description of The Healthy Start Study has been given in detail previously [15].

Recruitment of participants to the Healthy Start study took place between 2009 and 2011. Information on birth weight, pre-pregnancy BMI of the mother and maternal educational level was obtained from the Danish National Birth Register and administrative birth forms on all children born between 2004 and 2007 from 11 selected municipalities in the greater Copenhagen area [15]. A total of 5,902 children met the criteria and were randomized into an intervention group, a control group, or a shadow control group. After the randomization, children were identified and checked for eligibility in the Danish Central Person Registry (CPR). Children that had requested protection from participation in statistical or scientific surveys based on data delivered from the Danish Central Person Registry, had moved to another municipality, had emigrated or died, lived in an orphanage, or that had an unknown life status were not eligible and thus excluded (n = 2,180). A total of 3722 children complied with the inclusion criteria [15].

Children in the shadow control group were followed for their weight development in registers. However, in the present study, children from the shadow control group were excluded as there was no registry information or obtained measurements on either exposure or covariates.

For those randomised into the intervention or control groups, a letter was sent to the parents inviting them to participate in the study. A total of 635 children in the intervention and control groups attended the baseline examinations. A total of 290 children from the intervention or control group were excluded due to missing information on exposure, primary outcome (change in BMI z-score) or covariates. Thus, the final population size for the present study was 345 children, of which 34 were overweight at baseline. A detailed flowchart can be found in **S1 Fig.**

### Assessment of anthropometry

Change in BMI z-score from baseline to follow-up was the primary outcome. Body weight was measured to the nearest 0.1 kg using a mechanical weight or beam-scale type weight (Tanita BWB-800 or SV-SECA 710). The children were measured in underwear only and were asked to urinate before the weighing. If the child was using a diaper, a new diaper was put on before the weighing. Body height was measured barefoot or in stockings to the nearest 0.1 cm using a stature meter (Soehnle 5002 or Charter ch200P) [15]. BMI was calculated as weight in kilograms per height in meters squared. BMI z-scores were generated using the Lambda-Mu-Sigma (LMS) method, which summarises the changing distributions of the dependent variable (e.g. BMI) by the median, the coefficient of variation and skew expressed as Box-Cox power [16]. Using z-scores enables comparison of a measured BMI with adequate gender- and age-specific reference values [17]. It was chosen to apply a national reference of BMI z-score to the study population, and thus, a power transformation of 0.1 years of age was used [16].

Changes in waist circumference (WC), waist-hip ratio (WHR) and body fat percentage (BF%) were used as secondary outcomes. WC was measured to the nearest 0.5 cm midway between the lowest rib and the iliac crest. Hip circumference was measured to the nearest 0.5 cm, at the place where the circumference was the largest, seen from the frontal and medial angles. Both WC and hip circumferences were measured in triplicate and a mean was calculated. WHR was calculated based on these measurements [15].

Bioelectrical impedance was measured at a resistance of 50 kHz using a SEAC Multiple Frequency Bioimpedance Meter (model SFB3 and SFB2 version 1.0), RJL or Animeter (BIA-101 and BIA-103). Moreover, skinfolds were measured in triplicate at triceps and subscapular on the left side of the child, using Harpenden Skinfold Caliper or Lange Skinfold Caliper. From this, fat free mass (FFM) was estimated using an equation described by Goran et al. in young children [18]:

$$\begin{array}{l} FFM\;(kg)\\ \;=\left[0,16\times (\frac{H^{2}}{R})\right]+(0,67\times weight)-(0,11\times triceps)\\ \;-(0,16\times subscapular)+(0,43\times sex)+2,41\;kg \end{array}$$

Where H^2^/R is height^2^/resistance in cm^2^/Ω, weight is body weight in kg, triceps and subscapular are skinfold thicknesses in mm, and sex = 0 for girls and 1 for boys. Fat mass was calculated by subtracting fat free mass from body weight. BF% was calculated by dividing fat mass by body weight.

### Assessment of strengths and difficulties questionnaire

A Danish single-sided version of the Strengths and Difficulties Questionnaire (SDQ) was used. SDQ is a brief questionnaire screening for behavioural problems [19]. It is validated and has a high test-retest reliability and has been used in several large studies [6]. SDQ was an integrated part of a large questionnaire completed by the parents at baseline [16]. The questionnaire contained about 25 items related to the child’s behaviour, some positive and others negative. These 25 items are divided into 5 scales (“Emotional symptoms”, “Conduct problems”, “Hyperactivity/inattention”, “Peer relationship problems”, and “Prosocial behaviour”) [20]. The scores within all categories except “Prosocial behaviour” were summed to a Total Difficulties score (SDQ-TD score) and used as an exposure variable. The scale on Prosocial Behaviour was not incorporated into the SDQ-TD score, as absence of prosocial behaviours differs conceptually from the presence of psychological difficulties [19]. Therefore, the Prosocial Behaviour score (SDQ-PSB score) was used as an exposure variable by itself. In the present study we used the English scoring syntax for pre-school children available from the SDQ-webpage [21]. The exposure variables were included both as continuous and binary variables. The continuous SDQ-TD score variable ranged from 0–40 and SDQ-PSB score ranged from 0–10. For the binary SDQ-TD score and SDQ-PSB score variables, a category consisting of children above the 90^th^ percentile and a category of the remaining children was generated. This categorization was selected as a previously published cross-sectional study from the Healthy Start study suggested a higher BMI z-scores among children with an SDQ-TD score above the 90^th^ percentile [11].

### Covariates

The parents completed a large questionnaire on life style and demographic factors at baseline and follow-up [15]. Based on this questionnaire, the following variables were included as covariates. The mother’s physical activity level in leisure time over the past year with response options in 4 categories: 1) “*Regular and vigorous physical activity several times a week”*, 2) “*some physical activity”* (cycling to work, performing heavy gardening or similar a minimum of 4 hours per week), 3) *“light exercise”* (e.g. walking or light gardening a minimum of 4 hours per week), and 4) “*sedentary activities”* (e.g. reading or watching television). In the questionnaire the mother’s height and weight was self-reported and BMI was calculated based on this information. Maternal level of completed education was reported in 9 categories. Due to very few individuals in some of the groups, we recoded it into 4 categories: 1) *“Primary and lower secondary”*, *“upper secondary”*, *“one or more short courses”*, *“skilled worker”*, 2) *“short-term further education* [< 3 years]” or “*medium- term further education* [3–4 years]”, 3) *“long-term further education* [< 4 years]”, *“research level”* and 4) *“others”*. The parents were asked whether their child likes to be physically active with 5 possible answers. For this study, the 5 possible answers were recoded into the following 3 categories: 1) *“The child never thinks it is fun being physically active”* or *“the child rarely thinks it is fun being physically active”* or *“the child sometimes thinks it is fun to be physically active”*, 2) *“the child usually thinks it is fun to be physically active”*, and 3) *“the child always thinks it is fun to be physically active”*.

Information on the child’s total energy intake (MJ/day) was calculated based on a 4-day dietary record (Wednesday-Saturday) completed by parents, applying a picture book as guidance in reporting portion sizes. Finally, we included information on child sex, baseline age (years), and intervention status (intervention or control).

### Ethics

The Healthy Start study complied with the Helsinki declaration and approved by the Danish Data Protection Agency regarding participant's data protection (j. no. 2015-41-0530). The Scientific Ethical Committee of the Capital Region in Denmark decided that The Healthy Start Study was not a bioethics project and as a result did not need approval from the Danish Bioethics Committee (j.no. H-A-2007-0019). Written informed consent to use the collected data for research purposes was obtained from all parents of the participants [15].

### Statistical methods

Linear regression was used to examine the associations between SDQ-TD score and SDQ-PSB score and changes in BMI z-score and secondary outcomes (body fat percentage, WC and WHR) during follow-up. The statistical analyses were conducted in a stepwise manner using the following structure:

- Model 1: A crude model including information of exposure at baseline, change in outcome between baseline and follow-up and baseline measure of outcome.
- Model 2: Adjusted model with added information on potential baseline confounding factors (child age, BMI z-score, sex, intervention status, the physical activity level of the child, the physical activity level of the mother, maternal education and maternal BMI).
- Model 3: Same covariates as model 2, but with additional adjustment for the children's total energy intake at baseline to assess whether associations were mediated by calorie intake.

Model controls (i.e., investigating linearity of the relationship between outcomes and predictors, consistency with a normal distribution of the residuals, and variance homogeneity) were performed for the fully adjusted models. In the analyses of the primary outcome, possible effect modification by sex, intervention status or baseline level of outcome was examined by adding product terms to the models.

All statistical tests were two-tailed with a significance level at 0.05. Analyses were performed using Stata SE 14 (StataCorp LP, College Station, TX, USA; www.stata.com).

### Sensitivity analyses

Body fat percentage is a relative measure and thus changes in absolute values of lean and fat mass may occur independently of changes in the body fat percentage. Thus, as a sensitivity analyses, we further explored the associations between SDQ-scores and subsequent changes in fat mass index $\left[\frac{fat\;mass\;(kg)}{{height\;(m)}^{2}}\right]$ and fat free mass index $\left[\frac{fat\;free\;mass\;(kg)}{{height\;(m)}^{2}}\right]$.

## Results

Characteristics for children’s baseline SDQ-scores, adiposity measures at baseline and follow-up in addition to baseline covariates are shown in **Table 1**. As shown in the table, the median SDQ-TD was 6 (5–95 percentiles: 1; 13) and the median SDQ-PSB was 8 (5–95 percentiles: 5; 10) with almost identical values among boys and girls.

**Table 1:** Study characteristics of 345 children from the "Healthy Start" study.

	Total (n = 345)^1^	Boys (n = 193)	Girls (n = 152)
Children's age (years)	4.0 (2.5; 5.7)	4.1 (2.5; 5.7)	4.0 (2.4; 5.7)
**Exposure**			
SDQ-TD [1–4]	6 (1; 13)	6 (1; 13)	6 (2; 13)
SDQ-PSB [5]	8 (5; 10)	8 (4; 10)	8 (5; 10)
**Primary outcome**			
BMI z-score (baseline)	0.3 (-1.1; 1.8)	0.3 (-1.1; 1.8)	0.3 (-1.2; 1.8)
BMI z-score (follow-up)	0.4(-1.0; 1.8)	0.4 (-1.0; 1.8)	0.4 (-1.1; 1.7)
**Secondary outcomes**			
Body fat percentage (%) (baseline)	22.1 (7.6; 32.6)	20.0 (2.4; 30.9)	24.0 (9.6; 33.1)
Body fat percentage (%) (follow-up)	20.4 (8.0; 31.4)	17.7 (6.4; 28.0)	24.4 (14.7; 34.4)
Waist circumference (cm) (baseline)	52.3 (47.3; 57.7)	52.5 (48.0; 57.7)	52.2 (46.2; 58)
Waist circumference (cm) (follow-up)	53.2 (48; 59.3)	53.7 (48.3; 59)	52.4 (47; 59.7)
Waist-hip ratio (baseline)	1.0 (0.9; 1,0)	1.0 (0.9; 1.1)	0.9 (0.8; 1.0)
Waist-hip ratio(follow-up)	0.9 (0.8; 1.0)	0.9 (0.8; 1)	0.9 (0.8; 1.0)
**Covariates**			
Maternal physical activity (%)			
Sedentary activities	1.7	2.6	0.7
Light exercise	38.6	38.3	38.8
Some physical activity	49.9	51.8	47.4
Regular and vigorous physical activity	9.9	7.3	13.2
Education level (%)			
Low (less than short-term further education)	19.7	17.6	22.4
Short and medium-term further education	55.1	54.9	55.3
Long-term further education	24.1	26.4	21.1
Other	1.2	1.0	1.3
Child physical activity level (%)			
Never thinks it is fun being physically active	6.7	7.8	5.3
Usually thinks it is fun to be physically active	47.3	45.6	49.3
Always thinks it is fun to be physically active	46.1	46.6	45.4
Maternal BMI (kg/m^2^)	25.6 (20.1; 39.0)	24.9 (19.7; 39.0)	26.7 (20.6; 40.7)
Children’s total energy intake (kJ)	4,9 (3,3; 6,8)	5.0 (3,3; 6,8)	4,7 (3,2; 6,6)

Results presented as median and 5–95 percentiles unless stated otherwise.

^1^ The analyses of secondary outcomes are based on a reduced number of children: body fat percentage (n = 202), Waist circumference (n = 295), Waist-hip ratio (n = 291).

The association between SDQ-TD and SDQ-PSB scores at baseline and subsequent changes in BMI z-score and secondary outcomes are shown in **Table 2.** Neither the crude (model 1) nor the two adjusted models (models 2 and 3) showed significant associations between baseline SDQ-TD score and changes in BMI z-score, body fat percentage, WC, waist-hip ratio from baseline to follow-up (all p-values > 0.23). However, results from crude and adjusted models showed direct associations between baseline SDQ-PSB score and changes in BMI z-score from baseline to follow-up. Moreover, models 2 and 3 showed almost identical results (β: 0.040 [95% CI: 0.010; 0.071, p = 0.009] and 0.040 [95% CI: 0.010; 0.071, p = 0.009], respectively). We found no associations between baseline SDQ-PSB score and any of the body composition or fat distribution measures (all p > 0.17).

**Table 2:** Association between SDQ-TD score and SDQ-PSB score at baseline and subsequent change in BMI z-score and secondary outcomes.

	SDQ-TD			SDQ-PSB		
	β	95% CI	P-value	β	95% CI	P-value
**BMI z-score (n = 345)**						
Model 1^1^	0.009	(-0.005; 0.023)	0.23	0.038	(0.009; 0.068)	0.010
Model 2^2^	0.003	(-0.011; 0.018)	0.65	0.040	(0.010; 0.071)	0.009
Model 3^3^	0.004	(-0.011; 0.018)	0.63	0.040	(0.010; 0.071)	0.009
**Body fat percentage (n = 202)**						
Model 1	0.090	(-0.159; 0.340)	0.48	0.393	(-0.167; 0.952)	0.17
Model 2	0.068	(-0.173; 0.309)	0.58	0.351	(-0.190; 0.893)	0.20
Model 3	0.069	(-0.173; 0.310)	0.58	0.348	(-0.196; 0.892)	0.21
**Waist circumference (cm) (n = 295)**						
Model 1	0.016	(-0.049; 0.082)	0.62	0.054	(-0.094; 0.202)	0.47
Model 2	0.011	(-0.057; 0.080)	0.75	0.052	(-0.102; 0.205)	0.51
Model 3	0.010	(-0.058; 0.079)	0.77	0.053	(-0.101; 0.206)	0.50
**Waist-hip ratio (n = 291)**						
Model 1	-0.0003	(-0.001; 0.0007)	0.59	-0.0001	(-0.002; 0.002)	0.92
Model 2	-0.0003	(-0.001; 0.0007)	0.54	0.0004	(-0.002; 0.003)	0.75
Model 3	-0.0003	(-0.001; 0.0007)	0.57	0.0003	(-0.002; 0.003)	0.76

Abbreviations: SDQ-TD, Strengths and Difficulties Total Difficulties score; SDQ-PBS, Strengths and Difficulties Total Difficulties score Prosocial Behaviour score; BMI z-score, Body Mass Index Score z-score

^1^Crude model including information of exposure, change in outcome and baseline measure of outcome.

^2^ Adjusted model with added information on potential confounding factors (child age, BMI z-score, sex, intervention status, the physical activity level of the child, the physical activity level of the mother, maternal education and maternal BMI).

^3^Same covariates as model 2, but with additional adjustment for the children's total energy intake.

Likewise, we found a higher gain in BMI z-score among children with a SDQ-PSB above the 90^th^ percentile compared with the remaining children (model 2 β: 0.157 [95% CI: 0.014; 0.299], p = 0,03), while no differences in secondary outcomes were observed, and no difference in any outcomes were observed when comparing children with an SDQ-TD above the 90th percentile with the remaining children (all p-values > 0,18) **(S1 Table)**.

Finally, no evidence of associations was observed in sensitivity analyses exploring the associations between SDQ-scores and subsequent changes in fat mass index and fat free mass index (all p >0.13), and we found no evidence of effect modification by sex, intervention status or baseline measure of BMI z-score in analyses of change in BMI z-score.

## Discussion

In a population of 345 children, aged 2 to 6 years, primarily with normal-weight, who were predisposed to overweight, we found a direct association between the SDQ-PSB score and changes in BMI z-score. However, a similar association was not found in relation to change in body composition or shape. Moreover, we found no evidence of an association between SDQ-TD score and subsequent changes in BMI z-score or any of the secondary outcomes.

We found no previous prospective studies investigating the association between SDQ and subsequent changes in adiposity, but several cross-sectional studies have been published, generally suggesting that behavioural difficulties are directly linked to obesity [9, 10, 12, 13]. Likewise, we have previously shown that children with an SDQ-TD score above the 90^th^ percentile had higher BMI z-scores at a cross-sectional level [11], but our current longitudinal analyses did not suggest an association between SDQ-TD and development in adiposity. One prospective study has shown that obesity is associated with an undesirable development in mental health, as measured by SDQ [14]. Thus, in conjunction with our results, this may suggest that obesity has a negative impact on mental health of children, but not the other way around.

In terms of prosocial behaviour, cross-sectional results from Griffiths et al. suggests that girls with obesity at 3 years of age show more prosocial behaviour than girls with normal weight [22]. Though we found no evidence that this association was specific to girls, this result is consistent with the direct association between the SDQ-PSB score and changes in BMI z-score observed in our study. Moreover, our results suggest that this association was not driven by an increased relative fat accumulation, as no association was found in relation to change in BF% or fat and fat-free mass index. It is also worth mentioning that, although statistically significant, the association between the SDQ-PSB score and change in BMI z-score observed in our study was quite weak, with a 0.04 gain in BMI z-score per additional unit higher SDQ-PSB, corresponding to a weight gain of approximately 0.05 kg for a boy with a baseline age of 4 years over a period of 1.3 years.

The present study had several strengths. We had information from a validated questionnaire used to assess mental and behavioural strengths and difficulties, as well as objectively measured information on body weight, composition and shape. We used BMI z-scores generated using the Lambda-Mu-Sigma method, which enables comparison of a measured BMI with adequate gender- and age-specific reference values [17] in addition to BF%. Moreover, it has been suggested that WHR is not optimal to assess obesity in children as the measure may be age dependent [23]. Thus, we also conducted analyses with WC only.

A prospective study design was used, which examined association directions, a strength compared to a cross-sectional design. Furthermore, we had questionnaire data on several lifestyle factors, allowing us to adjust for potential confounding.

However, our study also presents some limitations. The changes in primary and secondary outcomes measures were relatively small, and we found no evidence of association between SDQ-scores and secondary outcomes. This lack of significant findings could be a consequence of the relatively short follow-up time (1.3 years), and potential anthropometric consequences of children’s behavioral problems may take longer to manifest.

Although we had information from a relatively large group of children, we cannot rule out that we have overlooked associations due to lack of statistical power. Nevertheless, the confidence intervals were generally narrow, making it unlikely that clinically important associations were overlooked. Moreover, information on which parent (mother or father) completed the questionnaire was not obtained. Other studies have suggested differences in reported child problems, depending on whether the mother or father completed the questionnaire. The father may tend to report more problems and fewer strengths in the child than the mother [24]. However, this would most likely lead to a dilution of the associations, as it seems unlikely that the potential variation in reporting depends on the subsequent weight change.

As in most observational studies, we cannot exclude that unmeasured or residual confounding have affected our results. For example, parental smoking habits have often been included as a confounder in similar studies [8] [25], but parental smoking status was not measured at the baseline examinations. Likewise, we cannot reject that residual confounding from physical activity level covariate has affected our results. We adjusted for the parents' perceptions of whether their child liked to be physically active, which is not necessarily a precise measure of the actual amount of physical activity the child performed. However, a review from 2012 showed that motivation correlates well with physical activity [26]. Moreover, as we generally found quite similar estimates in the crude and the adjusted analyses, we do not expect residual confounding to be a major issue in this study.

Finally, this study was conducted in a selected population of healthy weight children predisposed to obesity, and results may not be generalizable to other groups. Moreover, our study population, like most other studies, comprised a well-resourced group including children generally with low SDQ-TD scores. Thus, the observed associations may not be generalized to children with a higher degree of behavioural problems.

In conclusion, our study suggests no association between the SDQ-TD score and changes in BMI z-score, body composition or body shape among 2 to 6 years old children predisposed to overweight. The SDQ-PSB score was associated with a small increase in BMI z-score. However, this association does not seem mediated by an increased relative fat accumulation, as no association was found in relation to change in BF% or body shape.

## Supporting information

S1 Fig Flowchart of study population. (DOCX)

S1 Table Association between SDQ-TD score and SDQ-PSB score at baseline and subsequent change in BMI z-score and secondary outcomes for high scores compared with the remaining scores. (DOCX)
