# Supplementary material for: Child behaviour and subsequent changes in body weight, composition and shape
Source: PLoS One. 2019 Dec 19;14(12):e0226003. doi: 10.1371/journal.pone.0226003 (PMC6922444; doi:10.1371/journal.pone.0226003)
Supplement: S1 Fig — (DOCX) [file pone.0226003.s001.docx]

**S1 Fig**. Flowchart of study population

Participants at baseline

**n = 1055**

Intervention group: **n = 320**

Control group **n = 315**

Shadow control group **n = 420**

*Excluded***n = 290**

Missing information on exposures **n = 54**

Missing information on primary outcome (change in BMI z-score) **n = 199**

Missing information on baseline covariates **n = 37**

Study population with complete data at baseline and follow-up

**n = 345**

Intervention group **n = 157**

control group **n = 188**

Participants at baseline

**n = 635**

Intervention group **n = 320**

Control group **n = 315**

*Excluded*
Shadow control group. Followed in registers (information on weight and height only)

**n = 420**
