# Supplementary material for: Child behaviour and subsequent changes in body weight, composition and shape
Source: PLoS One. 2019 Dec 19;14(12):e0226003. doi: 10.1371/journal.pone.0226003 (PMC6922444; doi:10.1371/journal.pone.0226003)
Supplement: S1 Table — (DOCX) [file pone.0226003.s002.docx]

| **S1 Table. Association between SDQ-TD score and SDQ-PSB score at baseline and subsequent change in BMI z-score and secondary outcomes for high scores compared with the remaining scores** | | | | | | |
| --- | --- | --- | --- | --- | --- | --- |
|  | SDQ-TD | | | SDQ-PSB | | |
|  | **β** | **95% KI** | **P-value** | **β** | **95% KI** | **P-value** |
| **BMI z-score (n=345)** |  |  |  |  |  |  |
| Model 1 | -0.031 | (-0.239; 0.176) | 0.77 | 0.127 | (-0.014; 0.269) | 0.08 |
| Model 2 | -0.072 | (-0.281; 0.137) | 0.50 | 0.157 | (0.014; 0.299) | 0.031 |
| Model 3 | -0.069 | (-0.279; 0.140) | 0.52 | 0.158 | (0.015; 0.300) | 0.030 |
| **Body fat percentage (n=202)** |  |  |  |  |  |  |
| Model 1 | -1.261 | (-4.941; 2.420) | 0.50 | 0.029 | (-2.446; 2.504) | 0.98 |
| Model 2 | -2.119 | (-5.510; 1.272) | 0.22 | -0.614 | (-2.974; 1.746) | 0.60 |
| Model 3 | -2.095 | (-5.512; 1.322) | 0.23 | -0.628 | (-2.996; 1.740) | 0.60 |
| **Waist circumference (cm) (n=295)** | |  |  |  |  |  |
| Model 1 | 0.615 | (-0.343; 1.572) | 0.21 | 0.321 | (-0.324; 0.967) | 0.33 |
| Model 2 | 0.603 | (-0.359; 1.564) | 0.22 | 0.437 | (-0.218; 1.092) | 0.19 |
| Model 3 | 0.601 | (-0.362; 1.564) | 0.22 | 0.448 | (-0.210; 1.106) | 0.18 |
| **Waist-hip ratio (n=291)** |  |  |  |  |  |  |
| Model 1 | 0.003 | (-0.012; 0.017) | 0.70 | -0.004 | (-0.013; 0.006) | 0.45 |
| Model 2 | -0.00008 | (-0.014; 0.014) | 0.99 | -0.002 | (-0.012; 0.008) | 0.69 |
| Model 3 | -0.00004 | (-0.014; 0.014) | 1,00 | -0.002 | (-0.012; 0.008) | 0.67 |
| *Abbreviations: SDQ-TD, Strengths and Difficulties Total Difficulties score; SDQ-PBS, Strengths and Difficulties Total Difficulties score Prosocial Behaviour score; BMI z-score, Body Mass Index Score z-score* | | | | | | |
| *Model 1: A crude model including only information of exposure and outcome.* | | | | | | |
| *Model 2: Adjusted model with added information on potential confounding factors (child age, BMI z-score, sex, intervention status, the physical activity level of the child, the physical activity level of the mother, maternal education and maternal BMI).* | | | | | | |
| *Model 3: Same covariates as model 2, but with additional adjustment for the children's total energy intake to assess whether associations were mediated by calorie intake.* | | | | | | |
